# Neural responses to peers moderate conversation-drinking associations in daily life

**DOI:** 10.1038/s41598-025-05846-9

**Published:** 2025-07-24

**Authors:** Mia Jovanova, Ovidia Stanoi, Christin Scholz, Bruce Doré, Danielle Cosme, Yoona Kang, Nicole Cooper, Zachary M. Boyd, Dani S. Bassett, Peter J. Mucha, David M. Lydon-Staley, Kevin N. Ochsner, Emily B. Falk

**Affiliations:** 1 School of Medicine, University of St. Gallen, St. Gallen, Switzerland; 2 Department of Management, Technology, and Economics, ETH, Zurich, Switzerland; 3 Annenberg School for Communication, University of Pennsylvania, Philadelphia, Pennsylvania, USA; 4 Annenberg Public Policy Center, University of Pennsylvania, Philadelphia, Pennsylvania, USA; 5 Faculty of Social and Behavioral Sciences, University of Amsterdam, Amsterdam, The Netherlands; 6 Desautels Faculty of Management, McGill University, Montreal, Canada; 7 Department of Psychology, The State University of New Jersey, Camden, New Jersey, USA; 8 Department of Mathematics, Brigham Young University, Provo, Utah, USA; 9 Department of Bioengineering, University of Pennsylvania, Philadelphia, Pennsylvania, USA; 10 Department of Electrical & Systems Engineering, University of Pennsylvania, Philadelphia, Pennsylvania, USA; 11 Department of Neurology, University of Pennsylvania, Philadelphia, Pennsylvania, USA; 12 Department of Psychiatry, University of Pennsylvania, Pennsylvania, Philadelphia, USA; 13 Department of Physics & Astronomy, University of Pennsylvania, Philadelphia, Pennsylvania, USA; 14 The Santa Fe Institute, Santa Fe, New Mexico, USA; 15 Department of Mathematics, Dartmouth College, Hanover, New Hampshire, USA; 16 Leonard Davis Institute of Health Economics, University of Pennsylvania, Philadelphia, Pennsylvania, USA; 17 Department of Psychology, Columbia University, New York City, USA; 18 Wharton Marketing Department, University of Pennsylvania, Philadelphia, Pennsylvania, USA; 19 Wharton Operations, Information and Decisions Department, University of Pennsylvania, Philadelphia,Pennsylvania, USA

**Keywords:** Functional neuroimaging, Health behavior, Alcohol use, EMA (ecological momentary assessment), Social influence, Social groups, Human behaviour, Social behaviour

## Abstract

Conversations shape future behaviors, particularly among young adults. However, young adults vary widely in their susceptibility to peer influence. What neural processes relate to this susceptibility? We examined whether activity in brain regions associated with social rewards and making sense of others’ minds relates to a common health behavior—drinking, following conversations about alcohol. We studied ten social groups of college students (*N* = 104 students; 4760 total observations) across two university campuses. We collected whole-brain fMRI data while participants viewed photographs of peers with whom they tended to drink at varying frequencies. Next, using ecological momentary assessment, we tracked alcohol conversations and drinking twice daily for 28 days. On average, talking about alcohol was associated with a higher likelihood of next-day drinking. Controlling for baseline drinking, participants who responded more strongly to peers with whom they drank alcohol more frequently—in brain regions associated with social rewards and mentalizing—showed a stronger, positive association between alcohol conversations and next-day drinking. Conversely, stronger neural responses to peers with whom they drank less frequently decoupled associations between alcohol conversations and next-day drinking. We conceptually replicate prior findings linking conversations and drinking in an observational, longitudinal setting and provide new evidence that neural responses to peers moderate links between alcohol conversations and drinking behavior among young adults.

**Supplementary Information:**

The online version contains supplementary material available at 10.1038/s41598-025-05846-9.

## Introduction

Humans are inherently social beings. Many of our everyday decisions, including what we eat, drink and buy, are shaped by the actions, beliefs, and emotions of other individuals^1–3^. This phenomenon—social influence—has been widely studied across many fields and under different operationalizations. One major pathway to influence involves interpersonal communication^4,5^. Online and offline conversations about health topics relate to a wide range of behaviors such as alcohol use^6–9^ dietary habits^2,10,11^ exercise^12^ and smoking cessation^13^particularly among young adults. However, young adults vary widely in how likely they are to change their behavior in response to health-related conversations^14^.

What kinds of psychological processes help explain associations between conversations and future behaviors? A window into the nature of these processes is offered by functional neuroimaging (fMRI), which can identify neural systems associated with affective and social cognitive processes, and their engagement across individuals^15^. To examine between-person differences in the links between conversations and future behavior, we drew on recent paradigms that combine fMRI with ecological momentary assessment (EMA)^16^. fMRI can capture real time insights into how individuals process social cues^17^ and integrate inputs into health behavior^18^ while EMA can track health behaviors in real-world settings, for example through repeated text message surveys^19^. In this study, we combined fMRI and EMA to examine how individual differences in neural responses to social cues—specifically, the faces of peers—relate to the likelihood of drinking following alcohol conversations, using lagged analyses^9^. We focused on alcohol use as a prevalent behavior that influences health and well-being, particularly among a key population of young adults^20^ and in college settings^21^.

Neuroimaging studies have suggested the key role of several brain systems in processing social cues and subsequent behavior, including the reward system^22,23^ and the mentalizing system^24–26^. The reward system, including the bilateral ventral striatum, dorsal striatum (i.e., caudate and putamen), and ventromedial prefrontal cortex, is implicated in tracking rewards, for example, social rewards (e.g., approval from friends), food, or monetary rewards^27^. Individual differences in reward activity to social cues consistently correlate with conformity; with stronger reward activity and connectivity associated with higher likelihood to conform across substance use^28^, risky driving^29^, food choices^30^ and mobile app rating contexts^24,31^. A theoretical perspective underlying these findings suggests that humans value alignment with others, which fosters social connections and shared experiencess^32,33^. According to this view, individuals who are more likely to expect, or experience drinking-related rewards (e.g., social approval) as reflected by stronger activity in the reward system^34^ may also be more likely to conform to local drinking norms, i.e., by showing a more positive association between alcohol conversations and future drinking.

Consistent with this perspective, college drinking is largely social^21,35^ and shaped by social learning, where young adults often conform to drinking behaviors that are normative within their peer group^36^. Many students experiment with alcohol in the presence of peers and report drinking due to a desire to conform^37^. In parallel, in the brain, peer influence is often modulated by reward processing^15^with evidence that stronger reward activity to anticipated social rewards moderates links between peer norms and risk-taking susceptibility among adolescents^28^. Consistently, we anticipated that individuals with stronger reward responses to drinking peers versus non-drinking peers—defined here as peers with whom one drinks frequently vs. rarely^2,35^—would show a more positive association between alcohol conversations and drinking, compared to those without these reward-related brain patterns.

In addition to the brain’s reward system, the mentalizing system plays a central role in inferring and predicting mental states, or what peers think and expect^38^. Key regions involved in the mentalizing system, include the dorsomedial prefrontal cortex (dmPFC), temporoparietal junction (TPJ), posterior superior temporal sulcus (pSTS), and posterior cingulate cortex (PCC)^26,39^. Numerous studies have linked stronger activity in mentalizing regions with increased conformity among both young adults and adolescents^24,25,31,40–42^. Individuals with stronger activity in mentalizing regions may be more likely to consider peer viewpoints as more influential inputs to health decision-making^34,44^. Building on this work, we reasoned that, within college drinking contexts, individuals who show stronger mentalizing activity to drinking (vs. non-drinking) peers, possibly indicative of sensitivity to peer drinking-related expectations, may also show a more positive association between alcohol conversations and future drinking. Together, previous work points to the value of considering brain activity in both the reward and mentalizing systems to better understand individual differences in how conversations relate to future behavior.

Peers play a key source of influence, and may influence alcohol use directly (i.e., through feedback and conversations) and indirectly (i.e., through one’s mental representations of what peers might think and expect)^43,44^. For example, mental representations of drinking vs. non-drinking peers (with whom one drinks often vs. rarely) may evoke different values or social rewards attached to alcohol use. Further, mental representations of drinking peers may indirectly make local drinking norms salient, even without explicit peer pressure^45,46^, thereby potentially moderating to alcohol conversations and drinking^47,48^.

In parallel, the brain spontaneously tracks information about peers, such as their beliefs, traits, and behaviors during passive face viewing. Existing knowledge and perceptions of peers can be spontaneously retrieved during passive face processing^49^ and social attributes, such as status, can be tracked by activity in neural regions associated with reward and mentalizing^17,50,51^. Such mental schemas allow people to store and activate information about others rapidly via cognitive shortcuts or heuristics^49^. It is possible that different mental schemas of peers, evoked during passive face-viewing, may also become more (vs. less) activated during conversations. Bridging these disparate areas of research, individual differences in brain activity to one type of social signal—a peer’s face—may evoke different alcohol-related norms, expectations, or anticipated rewards, depending on prior drinking interactions with a given peer^2,35^. Individuals with stronger mentalizing and reward-related brain activity to the faces of drinking (vs. non-drinking) peers may more readily evoke pro-drinking schemas and anticipated social rewards, thereby showing a more positive association between alcohol conversations and drinking.

With the above considerations in mind, we examined how individual differences in reward and mentalizing activity to faces of drinking vs. non-drinking peers relate to lagged temporal associations between alcohol conversations and next-day drinking. We combined fMRI, with real-world social group information, and twice-a-day EMA over 28-days, to capture conversations and drinking within students’ daily lives^9,19^. We focused on existing, on-campus social groups where drinking is common^52^ and exacts a significant toll on the health, intellectual, and social lives of students^53^. Drawing on prior work suggesting that individuals with stronger neural sensitivity to social cues are more susceptible to social influence^24,29,31,34^ we hypothesized that individuals with stronger reward and mentalizing activity to the faces of drinking (vs. non-drinking) peers, will show a higher likelihood of next-day drinking following alcohol conversations.

## Results

### Descriptives

Throughout the 28-day EMA period, 99 out of 104 participants (~ 95%) reported drinking at least once and 97 (~ 93%) and reported at least one alcohol conversation. The average number of drinking occasions was 7.69 over 28 days (median = 6; *SD* = 6.07; range = 0–28) and the average number of alcohol conversations was 9.46 (median = 8; *SD* = 7.59; range = 0–34). No participants reported having both zero drinking occasions and zero alcohol conversations. See Fig. S4 for alcohol and conversation sample distributions. Participants were highly compliant with the study protocol, responding to a median of 95% (53/56) of alcohol conversation prompts (*M* = 50.17; *SD* = 7.8; range = 13–56) and 96% (53.5/56) of EMA alcohol use prompts (*M* = 51.03; *SD* = 7.2; range = 18–56). In total, we collected 4760 EMA data points. We observed large individual differences in neural responses to peer faces, with grand mean centered raw units of neural responses in the reward system ranging from − 0.56 to 0.54 (median =−0.01, *SD* = 0.24) and mentalizing activity ranging from − 0.84 to 0.80 (median =−0.03; *SD* = 0.31). Correlations between key study variables can be found in Supplement B Table S10. See Fig. S4. for alcohol conversations and drinking occasion sample distributions.

### Neural responses to peer faces, alcohol conversations and alcohol use

We found that talking about alcohol was associated with an increased likelihood of next-day drinking (OR = 1.59, 95% CI [1.30–1.94], *p* <.001). We next tested whether including a neural index of activity to the faces of a drinking vs. non-drinking peers, as a moderator, would provide additional information about who is more (vs. less) likely to drink following alcohol conversations (and would improve the model fit as measured via Akaike information criterion [AIC]^54^ and chi-square tests). Specifically, we tested whether including an interaction term between alcohol conversation and brain activity to drinking (vs. non-drinking) peers would improve model fit relative to (i) a main effects model (alcohol conversation and brain activity to peers as separate predictors) and (ii) a null model (no information about alcohol conversations and brain activity). We repeated this comparison twice to consider brain activity in the reward and the mentalizing regions separately, as presented below.

### Reward and mentalizing ROIs

The two-way interaction model (reward activity*alcohol conversation) significantly improved model fit (AIC = 3697.4), compared to a main effects model for alcohol conversation and activity in reward-related regions (AIC = 3699.6) and to a null model (AIC = 3746.9). Chi-squared tests showed that the interaction model significantly improved fit over the main effects model (^2^(1) = 4.16, *p* =.041) as well as the null model (^2^(1) = 57.45, *p* = < 0.001). We observed similar patterns when considering mentalizing activity. The two-way interaction model (mentalizing activity*alcohol conversation) significantly improved model fit (AIC = 3695.7) compared to a main efforts model for alcohol conversation and mentalizing activity (AIC = 3699.5) and to a null model (AIC = 3746.9). The interaction model significantly improved fit over the main effects model (^2^(1) = 6.05, *p* =.014) and the null model (^2^(1) = 59.16, *p* = < 0.001). Together, considering individual differences in brain activity to drinking (vs. non-drinking) peer faces—in reward and mentalizing systems—explained additional variance in associations between alcohol conversation and next-day drinking.

### Neural responses to peer faces moderate the association between conversations and drinking

We tested two hypotheses about the interaction between brain activity to peers and alcohol conversations on next-day drinking. We predicted that stronger brain activity in reward and mentalizing systems to drinking (vs. non-drinking) peers would moderate the temporal association between talking about alcohol and next-day drinking, with individuals with stronger brain activity showing a more positive association between alcohol conversation and next-day drinking.

**Fig. 1:**
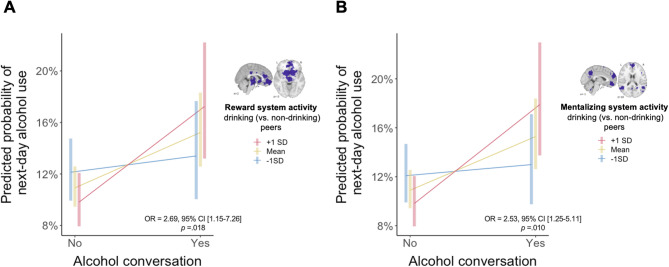
Neural responses to drinking vs. non-drinking peers moderate associations between alcohol conversations and next-day drinking.

Consistent with our first hypothesis, we observed a significant interaction between reward activity to drinking (vs. non-drinking) peers and alcohol conversations on next-day drinking (OR = 2.89, 95% CI [1.15–7.26], *p* =.024), such that stronger activity in the reward system was associated with higher likelihood of next-day drinking following alcohol conversations. Follow-up analyses showed that talking about alcohol was associated with higher likelihood of next-day drinking, and this association was stronger for individuals whose brains showed stronger (+ 1 *SD*) or near average reward-related activity to the faces of drinking peers (+ 1 *SD* reward activity: OR = 1.86, 95% CI [1.38–2.50], *p* <.001; near average reward activity: OR = 1.44, 95% CI [1.17–1.77], *p* =.006, Fig. 1). In other words, participants who discussed alcohol also showed a stronger likelihood of next-day drinking. Specifically, their likelihood of drinking the next day increased by about 4% (95% CI: 2–6%), from 11% (95% CI: 8–14%) to 15% (95% CI: 10–20%, Fig. 1). Additionally, stronger reward-related brain activity in response to drinking peers correlated with an increased likelihood of next-day drinking by approximately 7% (95% CI [4–8%]), from 10% (95% CI [7–14%]) to 17% (95% CI [11–22%], Fig. 1). By contrast, for participants whose brains showed stronger activity to non-drinking peers (−1 *SD*), talking about alcohol was not significantly associated with next-day drinking (−1 *SD* reward activity: OR = 1.12, 95% CI [0.82–1.52], *p* =.42; Fig. 1).

We observed similar patterns when considering brain activity in the mentalizing system. Consistent with our second hypothesis, we observed a significant interaction between mentalizing related-activity and alcohol conversations on next-day drinking, with stronger activity in the mentalizing system associated with increased likelihood of drinking (OR = 2.53, 95% CI [1.25–5.11], *p* =.010). Talking about alcohol was associated with increased drinking likelihood among individuals whose brains showed stronger (+ 1 *SD*) or near average mentalizing activity to the faces of drinking (vs. non-drinking) peers (+ 1 *SD* mentalizing activity: OR = 1.93, 95% CI [1.43–2.60], *p* <.001; near average mentalizing activity: OR = 1.45, 95% CI [1.18–1.78], *p* =.005, Fig. 1). Stronger mentalizing activity to drinking (vs. non-drinking) peers associated with an increased likelihood of drinking following alcohol conversations by approximately 7% (95% CI [4–10%]), from 9% (95% CI [7–12%]) to 16% (95% CI [11–22%], Fig. 1). By contrast, for people whose brains showed stronger activity to non-drinking (vs. drinking) peers, talking about alcohol was not significantly associated with next-day drinking (−1 *SD* mentalizing activity: OR = 1.09, 95% CI [0.80–1.47], *p* =.5912, Fig. 1). Together, these results suggest that individuals with stronger activity within the two hypothesized brain systems—reward and mentalizing—showed more positive associations between alcohol conversations and next-day drinking. See Tables 1 and 2 for full models with covariates.

Among participants who showed stronger or near average activity in (A) reward and (B) mentalizing systems to drinking peers, talking about alcohol was associated with higher likelihood of next-day drinking (+ 1 SD; pink line; mean; yellow line). Among participants with stronger activity to non-drinking peers (−1SD; blue line), talking about alcohol was not significantly associated with next-day drinking.

### Additional analyses

Sensitivity analyses and robustness checks are included in the Supplement B. These analyses include tests of whether neural responses to drinking (vs. non-drinking) peers moderate associations between alcohol conversations and next-day drinking when including outliers (Tables S1 & S2); removing covariates (Tables S3 and S4); controlling for self-reported peer ratings of peer closeness and peer liking (Tables S6 and S7); and applying zero-inflated negative binomial models, which separately model the count and likelihood of alcohol use occasions (Tables S8 and S9). We also explored the specificity of our results by replacing conversations about alcohol with conversations about non-alcohol topics, and neural responses tracking general perceived peer closeness (Tables S11 and S12) and conversational valence. Across all approaches, we observed parallel results such that stronger neural responses to drinking (vs. non-drinking) peers in the reward and mentalizing systems was associated with increased likelihood of drinking following alcohol conversations, and these relationships were specific to alcohol (vs. non-alcohol related) conversations.

## Discussion

Conversations play a key part in shaping health behaviors, and individuals vary widely in how they process and respond to such influences. To our knowledge, this is the first study to examine how individual differences in neural responses to real peers relate to associations between alcohol conversations and next-day drinking behavior. We sampled students at two Northeastern US college campuses who were members of existing social groups (e.g. sports teams and fraternities). Using fMRI, we observed brain activity while participants viewed photographs of the faces of peers within their social groups. Critically, faces showed peers with whom participants engaged in social drinking at varying frequencies. Next, using EMA, we tracked alcohol conversations and drinking behavior twice daily for 28 days. Controlling for individual differences in baseline drinking, we found that participants with stronger reward and mentalizing brain activity to the faces of drinking peers—with whom they drank more frequently—showed a stronger, positive association between alcohol conversations and next-day drinking. Conversely, participants with stronger brain activity to non-drinking peers—with whom they drank less frequently—showed no significant association between alcohol conversations and next-day drinking.

Our results are consistent with a growing body of work highlighting the role of mentalizing and reward processes in the brain in tracking relational status of others^17,50,51,55^ and social influence processes^11,12,30^. Our results extend existing literature in several important ways.

Prior neuroimaging studies have largely studied how individuals respond to social influence in controlled experimental conditions, for example by manipulating participant beliefs about perceived peer preferences for appetitive stimuli (e.g., to food or art) or by presenting normative ratings ostensibly made by anonymous others^25,30,56^. However, real-world health behavior processes are often more complex and nuanced, and knowledge about how young adults respond to naturalistic peer cues remains limited in substance use contexts^57^. Our study revealed that the associations between alcohol conversations and next-day drinking varied based on individuals’ mental representations of peers within their social groups. While we conceptually replicated the positive correlation between discussing alcohol and subsequent drinking in a naturalistic, longitudinal setting^9^we found that the direction and strength of this association varied significantly based on neural responses to peers in brain systems involved in motivational, affective, and social cognitive processing.

Consistently, social learning theory suggests that the nature of peer drinking relationships can shape drinking through learning mechanisms. For example, when peers frequently engage in drinking together, these interactions can reinforce similar behaviors and provide models for future alcohol consumption through observational learning. This often happens as young adults seek peer approval and try to avoid rejection^35^. In line with this view, it is possible that participants who responded more strongly to peers with whom they drank more frequently—in brain regions associated with social rewards and mentalizing—may be more likely to activate schemas related to expectations from peers or anticipated social rewards for drinking, thereby motivating a more positive association between alcohol conversations and next-day drinking, and a stronger vulnerability to conform to future drinking behaviors. When drinking with their peers, individuals may experience more positive social rewards, such as increased approval, acceptance, and feelings of belonging. Additionally, as individuals drink more with peers, it may lead to more alcohol-related conversations, creating a mutually reinforcing cycle.

With respect to specificity, sensitivity analyses revealed that the main interaction effect was specific to conversations about alcohol, versus other topics. Additionally, the main interaction between brain activity in response to drinking (versus non-drinking) peers remained robust after accounting for self-reported measures of peer closeness and liking. This finding suggests that the association between alcohol conversations and next-day drinking may be modified by how individuals evoke mental representations about peers in relation to alcohol, rather than general representations of peer closeness or liking. However, future research is needed to isolate this distinction more specifically. In our data, drinking frequency and peer closeness were correlated (*r =.*64); as well as drinking frequency and peer liking (*r =.*33; Supplement B, Table S5). Participants in our sample did not always report drinking with their closest or most liked peers. Thus, our data are consistent with the idea that links between alcohol conversations and drinking behavior may not be driven by general friendship alone. However, when examining neural responses to close vs. distant peers, we also observed a non-significant interaction on alcohol conversations and next-day drinking trending in the same direction, suggesting some potential overlap between peer-drinking frequency and peer-closeness at the neural level. Future research may fruitfully isolate how distinct aspects of peer relationships contribute to both alcohol conversations and drinking behavior.

A key complementary finding was that individuals with stronger reward and mentalizing activity to the faces of non-drinking peers (vs. drinking peers) showed no significant associations between alcohol conversations and next-day drinking, regardless of their baseline drinking levels; potentially suggesting a protective mechanism. It is possible that stronger reward and mentalizing activity to non-drinking (vs. drinking) peers may be more likely to activate schemas unrelated to drinking during alcohol conversations, thereby helping steer participants away from alcohol consumption. This finding aligns with previous research among adolescents, which suggests that perceptions of high peer support can modulate neural sensitivity to risk-taking and buffer risk taking behaviors^58^. This finding further motivates how to harness positive peer cues to possibly discourage unhealthy drinking habits among college students.

While our analyses are correlational, our findings may help generate future hypotheses for experimental strategies aimed at reshaping alcohol-related conversations and potentially shifting social norms around drinking. Prior experimental work has shown that changing the tone of alcohol-related conversations can reduce binge drinking intentions and influence perceived social norms, on average^59^. Additionally, text-based reminders that highlight the perspectives of non-drinking peers have been found to causally reduce alcohol use frequency among college students, on average^60^. Our study extends these findings by suggesting that individual differences in neural responses to peers—particularly in mentalizing and reward-related brain regions—correlate with conversation-drinking associations, and may perhaps help identify who is more vs. less likely to benefit from potential strategies that aim to decouple conversational influences on drinking.

Building on these associations, future studies could examine whether individuals who show stronger neural responsiveness to non-drinking peers (vs. drinking peers) are more receptive to interventions that redirect alcohol-related conversations. Just-in-time adaptive interventions, which deliver support during moments of heightened vulnerability—specific time windows when students are most susceptible to alcohol-related peer influence—and deliver targeted prompts (e.g., cues from non-drinking peers) during those periods represent a promising framework for testing these possibilities^61,62^. Thus, our findings generate specific hypotheses for future experimental work aimed at testing whether individual differences in neural responses to peers and temporal patterns in alcohol-related conversations can predict differential responsiveness to tailored interventions that seek to decouple peer influence from drinking behavior.

The current results should be interpreted considering the strengths and limitations of the study. Here, we combined fMRI and information about peers from existing social groups within an EMA design, embedded in individuals’ daily lives in a correlational, observational context. This multimodal approach allowed us to capture time-sensitive links between alcohol conversations and drinking behavior in everyday environments, thereby avoiding common biases that may arise when participants are asked to recall information about longer periods of time (e.g., alcohol consumed in the previous 30 days^63^). Also notable is the fact we employed the same study protocol across two college campuses, thereby enhancing the robustness of our findings. Although intensive assessment can raise data compliance concerns, we found minimal non-compliance among our sample. The intensive sampling approach produced high response rates, with ~ 95% median response rate over 28 days. This research also directly aligns with recent calls to connect laboratory findings on neural responses to real-world social contexts^57^ and to consider individual differences^64^ to better understand health behaviors like drinking.

With respect to limitations, our data cannot be used to generalize about samples beyond specific types of college students who are social drinkers (i.e., without alcohol dependence) who are part of social groups. Importantly, our sample includes students from two Ivy League institutions in the Northeastern United States, with a large portion of students coming from highly educated families, with high socioeconomic status. This limited demographic scope may restrict the findings’ generalizability to students from other educational backgrounds, regions, or socioeconomic statuses. The unique experiences and perspectives of students attending more diverse institutions and with more diverse backgrounds may not align with those of our sample, and consequently, the results may not accurately reflect the behaviors and attitudes of the broader college student population. We recruited students from pre-existing social groups on two campuses (and included groups in which 80% or more expressed interest). Although we controlled for group membership the non-independence may have confounded effects in unmeasurable ways. Additionally, 32% of participants were members of sports teams; however, we did not gather information on whether these teams were in season during the study. This temporal context could have influenced the students’ drinking patterns during the study.

A key limitation of this study is that we did not measure with whom participants talked about alcohol, who initiated these conversations, their involvement level, or conversation content, all of which could have an unmeasured influence on the association between alchol conversations and next-day drinking. Although we did measure conversational valence, i.e., how positive or negative each alcohol conversation was, our study did not indicate that valence interacted with brain activity and drinking behavior. In our sample, most alcohol conversations were positive, and the drinking norms on the studied college campuses favored drinking as normative. While the study was framed broadly around wellness, participants’ possible awareness of the focus on alcohol may have also possibly influenced their alcohol conversations, thereby reducing ecological validity. Future studies may more precisely capture the nature of alcohol conversations, potentially through the use of natural language processing and passive mobile sensing.

It is important to note that the main interaction finding—specifically the relationship between neural responses to peers, alcohol conversations, and next-day drinking—should not be interpreted as causal. Our data show that neural responses to peers are correlated with different levels of drinking after alcohol conversations. This interaction represents a correlational association rather than a direct cause-and-effect relationship, and does not imply that conversations about alcohol or differences in brain activity directly cause increased drinking. It is plausible that alcohol conversations and drinking may be mutually reinforcing. For example, positively discussing alcohol could lead to increased drinking, while drinking may stimulate more positive conversations about alcohol, thereby creating a feedback loop. However, in our data, we observed a unidirectional pattern: neural responses to peers specifically moderated how conversations about alcohol relate to next-day drinking, but the reverse was not supported. That is, neural responses to peers did not significantly moderate how drinking behavior correlates with next-day conversations about alcohol. This asymmetry is consistent across both hypothesized brain systems—the reward and mentalizing systems. Specifically, individuals with varying neural responses to peers did not show different associations between alcohol use and next-day conversations about alcohol in either the reward system [OR = 1.49, 95% CI [0.36–6.11], *p* =.583] or the mentalizing system [OR = 1.34, 95% CI [0.50–3.64], *p* =.562]. These findings further underscore the correlational nature of the observed effects. To isolate causal effects between alcohol conversations and subsequent drinking behaviors, future experimental studies are needed that manipulate and monitor the content of these discussions. Future studies may also employ hyperscanning paradigms during peer conversations to validate the brain mechanisms engaged in near-real time.

Notably, in our data, individual differences in neural responses to the faces of drinking (vs. non-drinking) peers moderated the incidence of drinking episodes but not the specific amount consumed when drinking, as shown in Supplement B Tables S8 and S9. One possibility is that neural responses to drinking vs. non-drinking peers may be more strongly correlated with the likelihood of initiating drinking episodes than with the amount consumed during those episodes—which may be subject to additional factors not considered here. Another possibility is in our current sample of social drinkers, we had insufficient variability in the number of drinks per occasion to detect differences. As such, future work could fruitfully explore how individual differences in brain activity to different peers relates to health decision making across various behavioral contexts.

In sum, the present study, is the first to our knowledge, to combine fMRI, with information about real social groups, and EMA to examine how individual differences in neural responses to peers relate to associations between alcohol conversations and next-day drinking. Individuals whose brains showed stronger responses to faces of drinking vs. non-drinking peers—in systems related reward and mentalizing—showed a more positive association between alcohol conversations and next-day drinking. This work highlights that the ways individuals’ brains gauge motivational relevance of social connections may be associated with drinking following alcohol conversations, or may provide a protective buffer, depending on mental representations of different peers. Specifically, the present data suggest that neural responses to the faces of peers with varying drinking interactions may serve as indicators of broader susceptibility to alcohol conversations and drinking among this sample of college students. Future work may validate this neural index across more representative samples and integrate it in interventions that embed positive peer influence to promote healthy behaviors.

## Methods

### Data

We use data from the Social Health Impact of Network Effects Study (SHINE), a multimodal, multisite project designed to provide insight into mind, body, and community relationships among social groups of young adults (see Refs^65–71^). All research, methods, and study protocols were approved by the Human Subjects Electronic Research Application (HSERA) Institutional Review Board (IRB) at the University of Pennsylvania and were acknowledged by the Human Research Protection Office of the Department of Defense. All research, methods, and study protocols were conducted in accordance with the IRB at the University of Pennsylvania and the Human Research Protection Office of the Department of Defense.

Participant recruitment and sample characteristics.

Students in two urban universities who belonged to on-campus groups (e.g., sports teams, arts groups, Greek life, etc.) were invited to participate. We recruited groups where more than 80% of the group expressed interest to participate, with the goal of facilitating data collection from many social partners in the group. At baseline, each participant was invited to upload a photo of themselves to build a pool of stimuli for the face viewing fMRI task. We collected 588 photos of peer faces across 24 social groups.

The current study includes 104 participants (*M*_age_=20.56 years, *SD*_age_=1.72), from the SHINE study, who completed a baseline online survey, an fMRI visit and a post-scan survey, and a 28-day EMA assessment. The sample comprised students from 10 social groups (mean group size = 42.36 students; *SD* = 21.38, median = 32). Participants reported the following gender and racial/ethnic identities: 63 women, 40 men, 1 other; 57 white, 32 Asian, 2 Black, 5 Latino/a, 8 Other/or more than one identity. Among the participants, 32% participated as part of a sports team, 48% as part of a performing arts group, 17% as part of Greek life, and 3% as another type of group. From these students, 24% identified as freshmen, 17% as sophomores, 28% as juniors, 29% as seniors, and 0.2% as other. Only 8% reported being international students. Regarding family income, 34% of participants reported a family income of $200,000 or more; 28% reported a family income between $100,000 and $199,999; 14% reported $75,000 to $99,999; 11% reported $50,000 to $74,999; 6% reported $20,000 to $34,999; 5% reported $35,000 to $49,999; and 3% indicated an income below $19,999. In terms of maternal education, 33% of mothers held a Bachelor’s degree; 35% a Master’s degree; 15% a Ph.D. or equivalent (M.D., J.D., etc.); 6% associate or professional degree; 4% attended some college; 7% completed high school or GED; and 1% had some high school education. For paternal education, 29% of fathers held a Master’s degree; 28% a Ph.D. or equivalent; 21% a Bachelor’s degree; 10% completed high school or GED; 6% an associate degree; 4% attended some college; and 2% had some high school education.

Among the participants, 32% participated as part of a sports team, 48% as part of a performing arts group, 17% as part of Greek life, and 3% as another type of group. From these students, 24% identified as freshmen, 17% as sophomores, 28% as juniors, 29% as seniors, and 0.2% as other. Only 8% reported being international students. Regarding family income, 34% of participants reported a family income of $200,000 or more; 28% reported a family income between $100,000 and $199,999; 14% reported $75,000 to $99,999; 11% reported $50,000 to $74,999; 6% reported $20,000 to $34,999; 5% reported $35,000 to $49,999; and 3% indicated an income below $19,999. In terms of maternal education, 33% of mothers held a Bachelor’s degree; 35% a Master’s degree; 15% a Ph.D. or equivalent (M.D., J.D., etc.); 6% associate or professional degree, 4% attended some college, 7% completed high school or GED, and 1% had some high school education. For paternal education, 29% of fathers held a Master’s degree, 28% a Ph.D. or equivalent, 21% a Bachelor’s degree, 10% completed high school or GED, 6% had an associate degree, 4% attended some college, and 2% had some high school education. See ‘Supplement A Recruitment’ for more details and Fig. S1 for participant exclusion and see Ref^65^. for further details on study protocol and procedures. All participants provided written informed consent and were paid for their participation.

### Measures and tasks

#### fMRI face-viewing task

To measure neural responses to peer faces, we used a task adapted from Refs^17,50,51^. In the scanner, participants viewed photographs of peers from their on-campus social group. The task stimuli were prepared from peer photographs collected during the baseline survey. Group members were asked to face the camera, with a neutral expression, and to have no objects in the background. Photographs were inspected manually by researchers for quality and cropped and converted to grayscale with equal luminance, for standardization. See Ref^65^. for details on task development and stimuli selection.

The face-viewing task implemented a rapid event-related design across two runs. During each run, participants viewed three trial types: faces of peers who were part of their on-campus social group (*M* = 25 unique peer faces; *SD* = 3; range = 18–27), their own face, and a red dot in the center of the screen (control images), appearing one at a time. Each face appeared on the screen 6 times (3 times per run) in a randomized order. All trials were presented for one second, followed by a jittered fixation cross (*M* = 5.5s, *SD* = 2.8). To ensure engagement during the task, participants were instructed to press a button each time they saw a red dot on the screen (∼10% of total presentations), using a five-button box. See ‘Supplement A Fig. S2’ for task visualization. The task was presented using PsychoPy (Version v3.0.0b11^72^. Following the scan, participants reported on how frequently they drank with each of their peers featured in the face-viewing task using a 9-point scale (1–9) in addition to other measures that probed dimensions of individual variation that were beyond the scope of the current report^65^.

#### Ecological assessment of conversations and drinking

Throughout the 28-day EMA period, participants received two survey prompts per day via the LifeData mobile app (www.lifedatacorp.com*)*: in the morning (8am) and in the evening (6pm). Surveys assessed alcohol-related behaviors in addition to other measures such as craving and mood not reported here^65^.

#### Alcohol conversations and drinking occasions in situ

To measure alcohol conversations, participants were asked: “Since the (EVENING/MORNING) survey, have you talked to someone about alcohol?” Participants answered using a “No/Yes” response option. To measure alcohol use, participants were asked: “Since the EVENING/MORNING survey, have you consumed any alcohol?” Participants answered using a “No” or “Yes” response option. Both items were measured twice a day, during the morning and evening EMA. For follow-up alcohol use measures, see ‘Supplement A Follow-up measures’ and for analyses with these measures see Supplement B Tables S8 and S9 and Supplement B (pp. 22–23).

#### Baseline measures

During the initial online survey, participants reported on demographics including age, gender, and race/ethnicity, in addition to typical drinking frequency and drinking amount in the past 6 months prior to the study. The race/ethnicity variable indicated Asian, Black, Latino/a, white, and Other status. For wording and measurement of the baseline drinking measures see ‘Supplement A Alcohol use baseline measures’.

### fMRI data acquisition, modeling, and ROI analysis

#### fMRI data collection

Neuroimaging data were acquired on 3 Tesla Siemens Prisma scanners equipped with a 64-channel head coil. High-resolution T1-weighted structural images were collected using an MPRAGE sequence (TI = 1,100ms, voxel size = 0.9 × 0.9 × 1 mm, 160 slices, field of view [FOV] = 256, repetition time [TR] = 1850ms, echo time [TE] = 3.91ms, flip angle = 8°). T2*-weighted functional images were also collected (voxel size = 3 × 3 × 3 mm, 42 slices, FOV = 70, TR = 1,000ms, TE = 30, flip angle = 62°). Additional, fMRI data pre-processing, modeling, and ROI analysis are reported in detail in Supplement A.

#### fMRI task modeling

Since we aimed to compute a neural index of responses to peer faces with varying drinking interactions, we extracted activity to peer faces as modulated by drinking frequency ratings, or how often each participant reported drinking with each individual peer. As such, we modeled the task using a parametric modulation design as implemented in SPM 12^73^. We constructed first level models for each participant that regressed periods of exposure to peer faces on mean centered, trial-by-trial differences in how frequently they reported drinking with each peer (range: 1–9). Models also included nuisance regressors that were modeled separately. These included periods of exposure to an individual’s own face, red dot, and five motion regressors described above. Thus, to compute a brain index that captures differences in responses to peer faces with varying drinking interactions, we extracted activity to peer faces as modulated by drinking frequency ratings, or how often each participant reported drinking with each of the peers whose face they viewed in the scanner (~ 25 peers).

#### ROI analysis

To define targeted regions of interest, we extracted two functionally defined maps from Neurosynth^74^. For reward-related activity, we used the search term ‘reward’ (922 studies; 30418 activations, *p* <.01, corrected). For mentalizing-related activity, we used the search term ‘mentalizing’ (151 studies; 6824 activations, *p* <.01, corrected). Next, to create a neural index of activity to peers with whom one drinks more vs. less frequently, we extracted mean parameter estimates from the parametric modulated viewing of peer faces within the (a) reward ROI and the (b) mentalizing (ROI). Greater values on these neural indices correspond to stronger activity to faces of peers with whom one engages in more (vs. less) frequent drinking activities (i.e., drinking vs. non-drinking peers). This procedure resulted in two values per participant that were used as individual difference measures of ‘reward’ and ‘mentalizing’ activity to faces of drinking (vs. non-drinking) peers, as a function of peer drinking interactions.

#### Data preparation

We took several steps to prepare the data prior to modeling. We aimed to model how alcohol conversations relate to prospective alcohol use, and we slid forward the alcohol use variable by one day (by two observations), as the questions were phrased to measure alcohol use since the previous survey, twice a day. That is, we considered whether today’s alcohol conversation correlated with tomorrow’s alcohol use^9^. Further, to attenuate the influence of outliers in the brain data, we winsorized activity in reward regions and mentalizing regions +/- 2 standard deviations from the mean, following outlier inspection. This cutoff applied to seven participants’ activity in reward-related regions and four participants’ activity in mentalizing regions. As a sensitivity test, we repeated all analyses including the outliers in the brain data and observed parallel results. See ‘Supplement B Tables S1 and S2’ for analyses including outliers.

#### Analysis plan

To examine whether individual differences in neural activity in reward and mentalizing regions to drinking vs. non-drinking peers moderates the association between talking about alcohol and next-day drinking, we estimated mixed-effects models separately for each brain system. The main predictor of interest was an interaction term of (a) engaging in a conversation about alcohol (Yes/No) and (b) a between-subject neural index (for either reward or mentalizing systems) that captured activity to drinking (vs. non-drinking) peers. The alcohol conversation variable was split into a within and between components following standards in the field (See Ref^75^. for example). Specifically, we created a person-level conversation variable by computing (a) the overall proportion of alcohol conversations that occurred across the EMA protocol and (b) a within-person daily variable indicating whether (1) or not (0) an alcohol conversation occurred at a given prompt. Our primary outcome was the likelihood of alcohol use, indicating whether (1) or not (0) alcohol was consumed at a given prompt^68,76^.

We controlled for the following covariates: baseline drinking frequency and amount in the past six months (to account for possible individual differences in overall drinking), age, gender, race/ethnicity, group membership, response rates, social weekend, time in study—as on average, participants reported drinking less over the study period, and condition effects as part of a larger intervention study^62^. Social weekend is defined as Thursday-Sunday (relative to rest of the weekdays) given higher drinking levels among college students during these days^77^. Two intervention variables were specified to include (1) between-person randomized condition assignment (mindfulness, perspective-taking, and control) and (2) within-person condition assignment (active vs. inactive week). Group membership is defined as belonging to one of ten on-campus social groups (i.e., sports clubs or fraternities). Results are robust to both the inclusion and exclusion of covariates (Supplement B tables S3 and S4). All numeric variables were grand-mean centered, and intercepts were allowed to vary randomly across people. Models did not converge when a random effect for alcohol conversations was included and, as such, a simpler model without this random effect estimate is presented.

To quantify the likelihood of a binary future drinking episode, we conducted multilevel binary logistic regression using the ‘glmer’ function from the lme4 package^78^. We specified the “bobyqua” algorithm to optimize model convergence, with “optCtrl = list(maxfun = 100000)”. As a sensitivity test, we repeated the same analyses using more complex multi-level hurdle models using glmmTMB^79^ and speficied a truncated negative binomial distribution. The zero inflated multi-level hurdle models are commonly used to model alcohol use with an excess of zeros and a skewed distribution of non-zero counts and to separately model two aspects of alcohol use: (1) whether alcohol was used at all (a binary outcome: yes or no) and (2) the number of alcohol use occasions among those who did consume alcohol (a count outcome)^18^. We observed parallel results using both analytical approaches. See Supplement B Tables S8 and S9 for results from multi-level hurdle models. All analyses were conducted in RStudio version 3.6.2^80^.

**Table 1:** Reward-related responses to drinking (vs. non-drinking) peers moderate associations between alcohol conversations and next-day drinking.

Alcohol conversation*reward activity and next-day drinking			
Fixed Effects	OR	95%CI	*P*
Intercept Alcohol conversation Reward activity Baseline drinking amount Baseline drinking frequency Gender Age Race Social group Social weekend (vs. weekdays) Proportion of alcohol conversations Alcohol responses Time in study Condition mindful (vs. control) Condition perspective (vs. control) Active week (vs. inactive) Alcohol conversation*reward activity	0.08 1.44 0.81 1.11 1.29 1.14 1.19 1.15 1.02 1.23 10.05 0.98 0.99 1.02 0.98 1.17 2.69	0.04, 0.17 1.17, 1.77 0.43, 1.54 0.04, 1.46 1.16–1.44 0.85, 1.52 1.10–1.29 1.02, 1.29 0.96, 1.08 1.04, 1.46 3.95, 25.58 0.96, 1.00 0.98, 0.99 0.72, 1.44 0.68, 1.40 0.94, 1.45 1.15, 7.26	< 0.001*** 0.001** 0.523 0.018 < 0.001*** 0.387 0.021* 0.557 0.557 0.018* < 0.001*** 0.102 < 0.001*** 0.920 0.896 0.166 0.024*
	ICC	SD	
Intercept Participant ID	0.07	0.50	

Note. 4760 observations. *p ≤.05, **p ≤.01, ***p ≤.001.

**Table 2:** Mentalizing-related responses to drinking (vs. non-drinking) peers moderate associations between alcohol conversations and next-day drinking.

Alcohol conversation*mentalizing activity and next-day drinking				
Fixed Effects	OR		95%CI	*P*
Intercept Alcohol conversation Mentalizing activity Baseline drinking amount Baseline drinking frequency Gender Age Race Social group Social weekend (vs. weekdays) Proportion of alcohol conversations Alcohol responses Time in study Condition mindful (vs. control) Condition perspective (vs. control) Active week (vs. inactive) Alcohol conversation*mentalizing activity		0.08 1.45 0.84 1.11 1.29 1.13 1.19 1.15 1.02 1.23 10.46 0.98 0.99 1.01 0.97 1.17 2.53	0.04, 0.18 1.18, 1.78 0.52, 1.37 1.04–1.19 1.16–1.44 0.85, 1.51 1.10, 1.29 1.02, 1.29 0.96, 1.08 1.04, 1.45 4.09, 26.79 0.96, 1.00 0.98, 0.99 0.71, 1.43 0.67, 1.40 0.94, 1.46 1.25, 5.11	< 0.001*** < 0.001*** 0.457 0.003 < 0.001*** 0.402 < 0.001*** 0.020 0.580 0.019* < 0.001*** 0.102 < 0.001*** 0.947 0.874 0.154 0.010*
	ICC		SD	
Intercept Participant ID	0.07		0.51	

Note. 4760 observations. *p ≤.05, **p ≤.01, ***p ≤.001.

## Electronic supplementary material

Below is the link to the electronic supplementary material.

Supplementary Material 1

## Data Availability

De-identified data and code to reproduce the main analyses are available on: https://github.com/miajov/brain-responses---peer-influence.

## Declarations

### Citation statement

We sought to proactively consider choosing references that reflect the diversity of the field in thought, form of contribution, gender, race, ethnicity, and other factors. First, we obtained the predicted gender of the first and last author of each reference (excluding software package citations) by using databases that store the probability of a first name being carried by a woman (https://github.com/dalejn/cleanBib). By this measure (and excluding self-citations to the first and last authors of our current paper), our non-software references contain 28% woman(first)/woman(last), 27% man/woman, 14% woman/man, and 31% man/man. This method is limited in that names used to construct the databases may not, in every case, be indicative of gender identity and cannot account for intersex, non-binary, or transgender people.

### Positionality statement

Mindful that our identities can influence our approach to science, the authors wish to provide the reader with information about our backgrounds. With respect to gender, when the manuscript was drafted, eight authors self-identified as women, one as non-binary, and four as men. With respect to race, 12 authors self-identified as white and one as Asian. With respect to engagement with college students, when this study was conducted, two authors were doctoral students who teach and/or mentor other students, three authors were postdoctoral researchers or research scientists who teach and/or mentor students, and eight authors were professors who teach and/or mentor students.
