# Supplementary material for: Neural responses to peers moderate conversation-drinking associations in daily life
Source: Sci Rep. 2025 Jul 24;15:26914. doi: 10.1038/s41598-025-05846-9 (PMC12290041; doi:10.1038/s41598-025-05846-9)
Supplement: Supplementary file 1 — Supplementary Material 1 [file 41598_2025_5846_MOESM1_ESM.docx]

**Supplementary Information**

The following document contains supplementary information for *Jovanova et al*. “Neural responses to peers moderate conversation-drinking associations in daily life”. Supplement A presents information on participant enrollment and study procedures. Supplement B includes sensitivity analyses and robustness checks.

**Supplement A**

Information on participant recruitment is adapted from Jovanova et al^1,^, and information on the neuroimaging session and fMRI preprocessing is taken from our study protocol, Ref^2^. Here we provide a summary for the interested reader.

**Recruitment**

Recruitment materials advertised a study titled “Social Health Impact of Network Effects Study (SHINE)” to undergraduate students who were members of on-campus social groups across two urban Northeastern universities in the United States. The study was advertised through flyers, university websites, and email communication. To reach students, researchers contacted group leaders and further employed a snowball sampling approach, such that participating students could share recruitment information with their peers who were members of on-campus social clubs or sports teams. For the current report, the data collection began on March 1st, 2019 and ended on March 31st, 2020, thus including time primarily on campus and as participants transitioned home at the start of the COVID-19 pandemic.

*
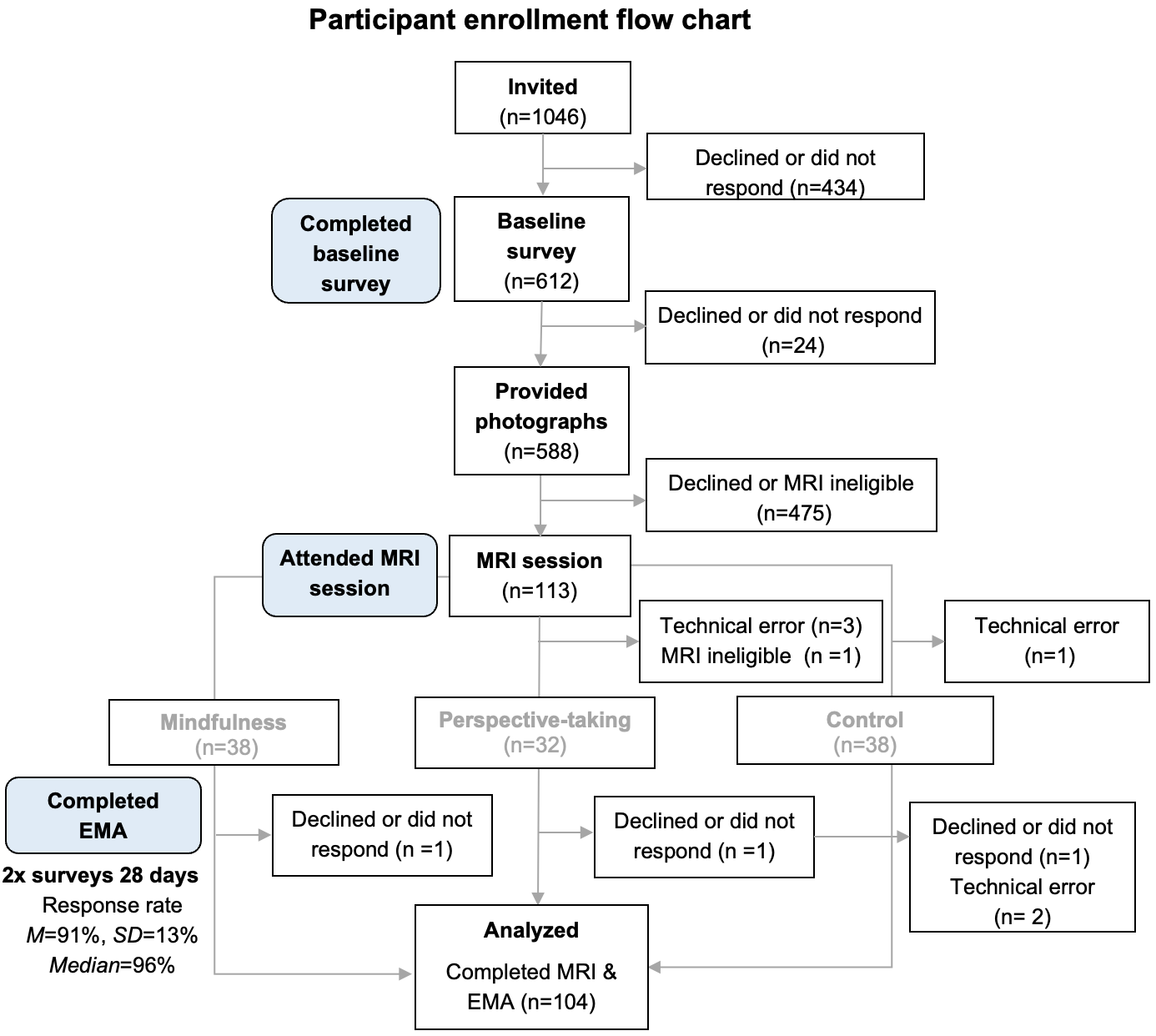
*

**Fig S1. Participant enrollment flowchart.**

Flow chart shows participant retention across three main study components: baseline survey, MRI session, and a 28-day EMA protocol. As part of a different study beyond the scope of the current report, participants were randomized into three conditions on how to respond to alcohol cues (mindfulness, perspective-taking, and control) (See Ref.^2^). See Supplement B Table S3 and S4 for analyses controlling for condition effects.

**
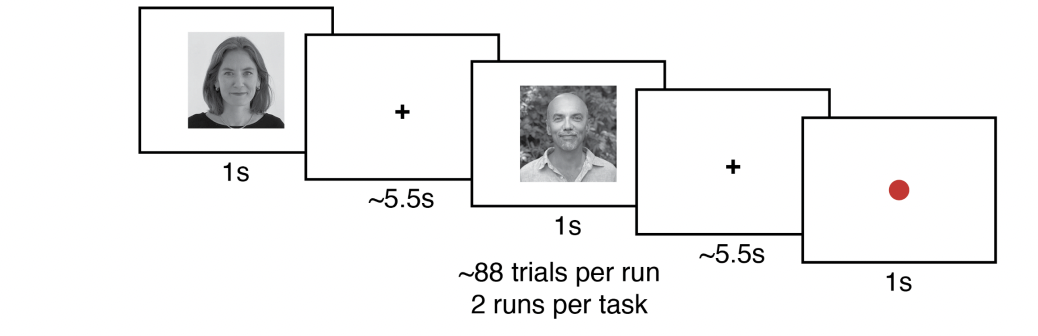
**

**Fig S2. Passive face viewing fMRI task.**

Design of the faces task. Participants viewed faces of their peers, themselves, or a control image (red dot), and pressed a button each time they saw the dot^2^. Please note the two photographs presented in Fig S2 show the faces of two of the authors as examples to illustrate the task design. These images do not show any identifiable participant information. The authors provided informed consent to have their faces included in the stimuli mockup.

**Faces task instructions**

The following text presents the verbatim task instructions for the fMRI task. Non-italicized fonts are researcher instructions and *italicized* fonts are instructions included in the PsychoPy scripts. Research assistants walked participants through the instructions and asked participants to complete several practice rounds on their own prior to beginning the fMRI task session.

| We’re going to be doing a task which will involve looking at pictures and pressing a button every time a red “O” appears on the screen. We are going to ask you to pay attention to the screen and press a button every time you see the red “O” appear on the screen.  *Please read and listen carefully, and let us know if you have any questions.*  *Each trial will start with a cross at the center of the screen (+). When you see this symbol, simply focus on its center.*  *Next, a photo will appear. You will see images of faces or a red “O”. You should focus on the screen. Press any button on the keypad when you see a red “O”. Do not press a button when you see a face.*  *You will have a few seconds to make your response. If three seconds pass and you haven't yet made a response, the computer will not record a response and will go on. To avoid this outcome, please keep your hand on the keypad and be ready to record a response.*  So I will walk you through some practice now… |
| --- |

**Face-viewing task pre-processing, modeling, and ROI analysis**

**fMRI preprocessing**. The anatomical and functional data were preprocessed using fMRIPrep (Version 20.0.6[^3^](https://paperpile.com/c/pU2wHm/Wx4n7), which is based on Nipype (Version 1.4.2[^4^](https://paperpile.com/c/pU2wHm/e87i);. The T1-weighted (T1w) image was corrected for intensity non-uniformity (INU) with N4BiasFieldCorrection[^5^](https://paperpile.com/c/pU2wHm/IriCq), distributed with ANTs 2.2.0[^6^](https://paperpile.com/c/pU2wHm/twcZB), and used as a T1w-reference throughout the workflow. The T1w-reference was then skull-stripped with a Nipype implementation of the ANTs brain extraction workflow, using OASIS30ANTs as target template. Brain tissue segmentation of cerebrospinal fluid (CSF), white-matter (WM), and gray-matter (GM) was performed on the brain-extracted T1w image using FAST (FSL 5.0.9[^7^](https://paperpile.com/c/pU2wHm/KVBtp)). Brain surfaces were reconstructed using recon-all (FreeSurfer 6.0.1[^8^](https://paperpile.com/c/pU2wHm/k4CRP)), and the brain mask estimated previously was refined with a custom variation of the method to reconcile ANTs-derived and FreeSurfer-derived segmentations of the cortical gray matter of Mindboggle[^9^](https://paperpile.com/c/pU2wHm/hQim3). Volume-based spatial normalization to one standard space (MNI152NLin2009cAsym[^10^](https://paperpile.com/c/pU2wHm/VIdPn)) was performed through nonlinear registration with antsRegistration (ANTs 2.2.0), using brain-extracted versions of both the T1w reference and the T1w template.

A reference volume and its skull-stripped version were generated using a custom methodology of fMRIPrep. A B0-nonuniformity map (or fieldmap) was estimated based on two echo-planar imaging (EPI) references with opposing phase-encoding directions, with 3dQwarp[^11^](https://paperpile.com/c/pU2wHm/Xy7zQ) with AFNI 20160207. Based on the estimated susceptibility distortion, a corrected EPI reference was calculated for a more accurate co-registration with the anatomical reference. The BOLD reference was then co-registered to the T1w reference using bbregister from FreeSurfer, which implements boundary-based registration[^12^](https://paperpile.com/c/pU2wHm/fTzUT). Co-registration was configured with six degrees of freedom. Head-motion parameters with respect to the BOLD reference (transformation matrices and six corresponding rotation and translation parameters) are estimated before any spatiotemporal filtering using mcflirt (FSL 5.0.9)[^13^](https://paperpile.com/c/pU2wHm/L65zV). BOLD runs were slice-time corrected using 3dTshift from AFNI 20160207[^11^](https://paperpile.com/c/pU2wHm/Xy7zQ). The BOLD time series were resampled onto their original, native space by applying a single, composite transform to correct for head-motion and susceptibility distortions. The BOLD time series were resampled into standard space, generating a preprocessed BOLD run in MNI152NLin2009cAsym space. All resamplings were performed with a single interpolation step by composing all the pertinent transformations (i.e., head-motion transform matrices, susceptibility distortion correction when available, and co-registrations to anatomical and output spaces). Gridded (volumetric) resamplings were performed using antsApplyTransforms (ANTs), configured with Lanczos interpolation to minimize the smoothing effects of other kernels[^14^](https://paperpile.com/c/pU2wHm/sgcWm). Non-gridded (surface) resamplings were performed using mri_vol2surf (FreeSurfer). Various confounds (e.g., framewise displacement, DVARS, and global signal) were also calculated for each TR and logged in a confounds file. The outputs from fMRIPrep were then manually quality checked to ensure adequate preprocessing.

Prior to first-level modeling, we generated motion regressors using an automated motion assessment tool. This tool is a predictive model that utilizes the confound files generated by fMRIPrep and classifies whether or not fMRI volumes contain motion artifacts. The classifier is applied to each participant’s task run and returns a binary classification indicating the presence or absence of motion artifacts for each volume. In addition, this tool transforms the realignment parameters into Euclidean distance for translation and rotation separately and calculates the displacement derivative of each. This yielded a total of five motion regressors for first-level modeling[^2^](https://paperpile.com/c/pU2wHm/nAIG). First, realignment parameters were transformed into Euclidean distance for translation and rotation separately, and we included the displacement derivative of each (resulting in four motion regressors). Another regressor of non-interest marked images with motion artifacts (e.g., striping) was identified via automated motion assessment[^15^](https://paperpile.com/c/pU2wHm/tgh9) and visual inspection. Following the application of this threshold, no task runs were excluded from further analyses. Data were high-pass filtered at 128s, and temporal autocorrelation was modeled using FAST[^16^](https://paperpile.com/c/pU2wHm/HWnM).

**Alcohol use baseline measures**

To measure typical alcohol use prior to data collection, participants responded to two questions which measured drinking frequency and drinking amount as part of an online survey, in addition to other individual difference measures beyond the scope of the current report. Responses to these baselines drinking measures were included as covariates in the main models reported in the manuscript.

**Drinking frequency.** “During the last 6 months, how often did you usually have any kind of drink containing alcohol? (By a drink we mean the equivalent of a 12 oz can or glass of beer, a 5 oz glass of wine, or a drink containing 1 shot of liquor)”. Response options included: “I never drank any alcohol in my life” = 1; “I did not drink alcohol in the last 6 months, but I did drink in the past” = 2; “1-2 times in the past 6 months” = 3; “3-5 times in the past 6 months” = 4; “2-3 times a month” = 5; “Once a month” = 6; “Once a week” = 7; “Twice a week” = 8; “3-4 times a week” = 9; “5-6 times a week” = 10; “Every day” = 11.

**Drinking amount.** “During the last 6 months, how many alcoholic drinks did you have on a typical day when you drank alcohol?” “0 drinks” = 0; “1 drink” = 1; “2 drinks = 2; “3-4 drinks” = 3.5; “5-6 drinks” = 5.5; “7-8 drinks” = 7.5; “9-11 drinks” = 10; “12 - 15 drinks” =13.5; “16-18 drinks” = 17 drinks; “19-24 drinks”= 21.5; “25 or more drinks” = 25.

**Follow-up measures.** Participants who responded ‘yes’ to having alcohol were also asked to enter the number of wine/beer/liquor beverages consumed since the last survey as part of a different study^1^. Participants who reported ‘yes’, to having an alcohol conversation were asked to report the valence of their most recent alcohol conversation since the previous survey (0, *negative* to 100, *positive*). In the present manuscript, we focus on the initial “No/Yes'' responses to alcohol use and alcohol conversations. Analyses with follow-up measures are included in Supplement B Tables S8 and S9.

Supplement B

**Interaction effects of brain activity and alcohol conversations on next-day drinking are robust to outlier inclusion.** We conducted an exploratory analysis including outlier observations in brain activity in reward and mentalizing regions to drinking (vs. non-drinking) peers. In both cases, we found that the interaction between reward/mentalizing activity and alcohol conversations on next-day drinking remained significant. Stronger activity in reward and mentalizing regions was associated with an increase in the likelihood of drinking following alcohol conversations [reward: OR = 2.73, 95% CI [1.22=6.12], *p=*.014; mentalizing: OR =1.88, 95% CI [1.09-3.24], *p* =.023]. See Tables S1 and S2.

**Table S1. Reward ROI model including outliers.**

| **Reward ROI*alcohol conversation effects on next-day drinking** | | | | |
| --- | --- | --- | --- | --- |
|  | |  | | |
| Fixed Effects | | OR | 95%CI | *p* |
|  | Intercept  Alcohol conversation  Reward activity  Baseline drinking frequency  Baseline drinking amount  Proportion of alcohol conversations  Time in study  Alcohol conversation*reward activity | 0.18  1.47  0.57  1.34  1.08  11.84  0.99  2.73 | 0.14, 0.22  1.19, 1.80  0.34, 1.08  1.19, 1.50  1.00, 1.16  4.36, 32.15  0.98, 0.99  1.22, 6.12 | <.001***  <.001***  .089  <.001***  .040*  <.001***  <.001***  .014* |
|  | | ICC | SD | |
| Intercept  Participant ID | | .10 | .59 | |

*Note.* 4760 Observations. **p* ≤ .05, ***p* ≤ .01, ****p* ≤ .001.

**Table S2. Mentalizing ROI model including outliers.**

| **Mentalizing activity*alcohol conversation effects on next-day drinking** | | | | |
| --- | --- | --- | --- | --- |
|  | |  | | |
|  | |  | | |
| Fixed Effects | | OR | 95%CI | *p* |
|  | Intercept  Alcohol conversation  Mentalizing activity  Baseline drinking frequency  Baseline drinking amount  Proportion of alcohol conversations  Time in study  Mentalizing activity*alcohol conversation | 0.18  1.48  0.71  1.34  1.07  11.73  0.99  1.88 | 0.14, 0.22  1.20, 1.81  0.49, 1.03  1.19, 1.51  1.00, 1.15  4.31, 31.92  0.98, 0.99  1.09, 3.24 | <.001***  <.001***  .072  <.001***  .052  <.001***  <.001***  .023* |
|  | | ICC | SD | |
| Intercept  Participant ID | | .10 | .59 | |

*Note.* 4760 Observations. **p* ≤ .05, ***p* ≤ .01, ****p* ≤ .001.

**Interaction effects of brain activity and alcohol conversations on next-day drinking are robust to covariate exclusion**. We conducted exploratory analyses to inspect the robustness of our interaction effects when removing all covariates. We performed two models for reward and mentalizing ROIs separately. We observed consistent effects, such that neural responses to peers in reward and mentalizing regions moderated the association between alcohol conversations and next-day drinking, with stronger activity to drinking (vs. non-drinking) peers associated with an increased likelihood of drinking following alcohol conversations [reward: OR = 2.59, 95% CI [1.03-6.53], *p=*.043; mentalizing: OR = 2.35, 95% CI [1.17, 4.72], *p* =.017]. See Tables S3 and S4.

**Table S3. Reward ROI model without covariates**

| **Reward activity*alcohol conversation effects on next-day drinking** | | | | |
| --- | --- | --- | --- | --- |
|  | |  | | |
| Fixed Effects | | OR | 95%CI | *p* |
|  | Intercept  Alcohol conversation  Reward activity  Alcohol conversation*reward activity | 0.12  1.73  0.60  2.59 | 0.10, 0.15  1.42, 2.12  0.26, 1.39  1.03, 6.53 | <.001***  <.001***  .234  .043* |
|  | | ICC | SD | |
| Intercept  Participant ID | | .18 | .85 | |

*Note.* 4760 Observations. **p* ≤ .05, ***p* ≤ .01, ****p* ≤ .001.

**Table S4. Mentalizing ROI model without covariates**

| **Mentalizing activity*alcohol conversation effects on next-day drinking** | | | | |
| --- | --- | --- | --- | --- |
|  | |  | | |
| Fixed Effects | | OR | 95%CI | *p* |
|  | Intercept  Alcohol conversation  Activity in mentalizing regions  Alcohol conversation*mentalizing activity | 0.12  1.74  0.64  2.35 | 0.10, 0.15  1.42, 2.13  0.34, 1.22  1.17, 4.72 | <.001***  <.001***  .174  .017* |
|  | | ICC | SD | |
| Intercept  Participant ID | | .18 | .86 | |

*Note.* 4760 Observations. **p* ≤ .05, ***p* ≤ .01, ****p* ≤ .001.

**Interaction effects of brain activity and alcohol conversations on next-day drinking are robust to self-reported peer closeness and liking ratings.** To account for possible confounding influence of peer liking and closeness on the main interaction, we replicated our main analyses, controlling for self-reported peer ratings of peer closeness and peer liking. See Table S5 for correlations between self-reported peer measures. We found consistent results such as individuals with stronger brain activity to drinking (vs. non-drinking) peers showed a more positive association between alcohol conversations and next-day drinking in both mentalizing and reward regions [mentalizing: OR=2.48, 95% CI [1.22-5.02], *p*=0.012; reward: OR=2.81, 95% CI [1.11-7.09], *p*=0.029], while controlling for individual differences in peer liking and peer closeness. This finding builds our confidence that the observed interaction effects may not be driven by individual differences in peer closeness and peer liking. See Tables S6 and S7 for full model results.

**Table S5. Correlations and Descriptive Statistics of Self-reported Peer Ratings.**

| **Variables** | **1** | **2** | **3** |
| --- | --- | --- | --- |
| **1. Perceived peer liking**  *How much do you like [peer name]?* | - |  |  |
| **2. Perceived peer drinking frequency**  *How often do you drink with [peer name]?* | 0.33 | - |  |
| **3. Perceived peer closeness**  *How close are you to [peer name]?* | 0.60 | 0.64 | - |
| **Mean** | 5.33 | 3.08 | 3.68 |
| **Standard Deviation**  **Range** | 1.92  0-8 | 2.37  0-8 | 2.32  0-8 |

*Note*: *N*=103. Each participant rated 25 peers (*SD* = 2.76) on average.

**Table S6. Reward ROI model controlling for peer closeness and peer liking.**

| **Reward activity*alcohol conversation and next-day drinking** | | | | |
| --- | --- | --- | --- | --- |
|  | |  | | |
| Fixed Effects | | OR | 95%CI | *p* |
|  | Intercept  Alcohol conversation  Reward activity  Baseline drinking amount  Baseline drinking frequency  Gender  Age  Race  Social group  Social weekend (vs. weekdays)  Proportion of alcohol conversations  Alcohol responses  Time in study  Condition mindful (vs. control)  Condition perspective (vs. control)  Active week (vs. inactive)  Peer liking  Peer closeness  Alcohol conversation*reward activity | 0.10  1.45  0.84  1.11  1.29  1.20  1.19  1.16  1.02  1.21  10.66  0.98  0.99  1.01  0.99  1.16  1.05  0.92  2.81 | 0.04, 0.27  1.18, 1.78  0.43, 1.63  1.04, 1.19  1.16, 1.44  0.88-1.65  1.10, 1.30  1.03-1.31  0.96, 1.08  1.02, 1.44  4.14, 27.48.  0.96, 1.00  0.98, 0.99  0.71-1.43  0.69, 1.42  0.93, 1.45  0.87, 1.26  0.74, 1.13  1.11, 7.09 | <.001***  .001**  .607  .002  <.001***  .254  <.001***  .015*  .539  .026*  <.001***  .124  <.001***  .960  .946  .186  .633  .419  .029* |
|  | | ICC | SD | |
| Intercept  Participant ID | | .07 | .50 | |

*Note*. 4711 observations. **p* ≤ .05, ***p* ≤ .01, ****p* ≤ .001.

**Table S7. Mentalizing ROI model controlling for peer closeness and peer liking.**

| **Mentalizing activity*alcohol conversation and next-day drinking** | | | | |
| --- | --- | --- | --- | --- |
|  | |  | | |
| Fixed Effects | | OR | 95%CI | *p* |
|  | Intercept  Alcohol conversation  Reward activity  Baseline drinking amount  Baseline drinking frequency  Gender  Age  Race  Social group  Social weekend (vs. weekdays)  Proportion of alcohol conversations  Alcohol responses  Time in study  Condition mindful (vs. control)  Condition perspective (vs. control)  Active week (vs. inactive)  Peer liking  Peer closeness  Alcohol conversation*reward activity | 0.10  1.46  0.86  1.12  1.29  1.20  1.19  1.16  1.02  1.21  11.10  0.98  0.99  1.00  0.98  1.17  1.05  0.92  2.48 | 0.04, 0.27  1.18, 1.79  0.52, 1.42  0.04, 1.20  1.15,1.44  0.87, 1.64  1.10,1.30  1.03, 1.31  0.96, 1.08  1.02, 1.42  4.28, 28.82  0.96, 1.00  0.98, 0.99  0.70, 1.42  0.68, 1.42  0.94, 1.45  0.87, 1.26  0.74,1.14  1.22, 5.02 | <.001***  .001**  .558  .002*  <.001***  .268  <.001***  .015*  .560  .028*  <.001***  .124  <.001***  .992  .921  .172  .638  .432  .012* |
|  | | ICC | SD | |
| Intercept  Participant ID | | .07 | .50 | |

*Note*. 4711 observations. **p* ≤ .05, ***p* ≤ .01, ****p* ≤ .001.

**Brain activity interactions effects on alcohol conversations and next-day drinking are specific to drinking occasions.** As an additional test, we repeated the same analyses presented in the main manuscript using multi-level hurdle models, an alternative analytic approach which separately models the count of drinks per drinking occasion in addition to the presence (vs. absence) of alcohol use occasions, and accounts for the skewness of the data. This analysis allowed us to (a) confirm that our results are robust to different analytic choices and (b) explore the extent to which the observed interaction effects are specific to likelihood of a drinking occasion (zero-inflated model) versus number of drinks per drinking occasion (conditional model). See Figure S4A for sample distribution of alcohol conversations and Figure S4B for sample alcohol use distribution.

We observed parallel results to those presented in the main manuscript, such that brain activity in reward and mentalizing ROIs, moderated the association between alcohol conversations and next-day drinking occasions, with stronger activity associated with an increased likelihood of drinking following alcohol conversations [reward: OR =0.34, 95% CI [0.13-0.86], *p=*.022; mentalizing: OR =0.38, 95% CI [0.19-0.78], *p* =.008]. Further, we observed no significant interaction between brain activity in reward and mentalizing ROIs and alcohol conversations on the number of drinks per occasion [reward: OR =1.90, 95% CI [0.80, 4.54], *p=*.147; mentalizing: OR =1.29, 95% CI [0.68-2.48], *p* =.435] See Table S8 and S9. Together, these results suggest that the observed interaction is specific to the likelihood of engaging in a drinking occasion versus the number of drinks consumed when drinking. See Table S10 for descriptives and correlations of key variables.

**Fig. S4. Alcohol conversations and drinking occasion sample distributions.**

**
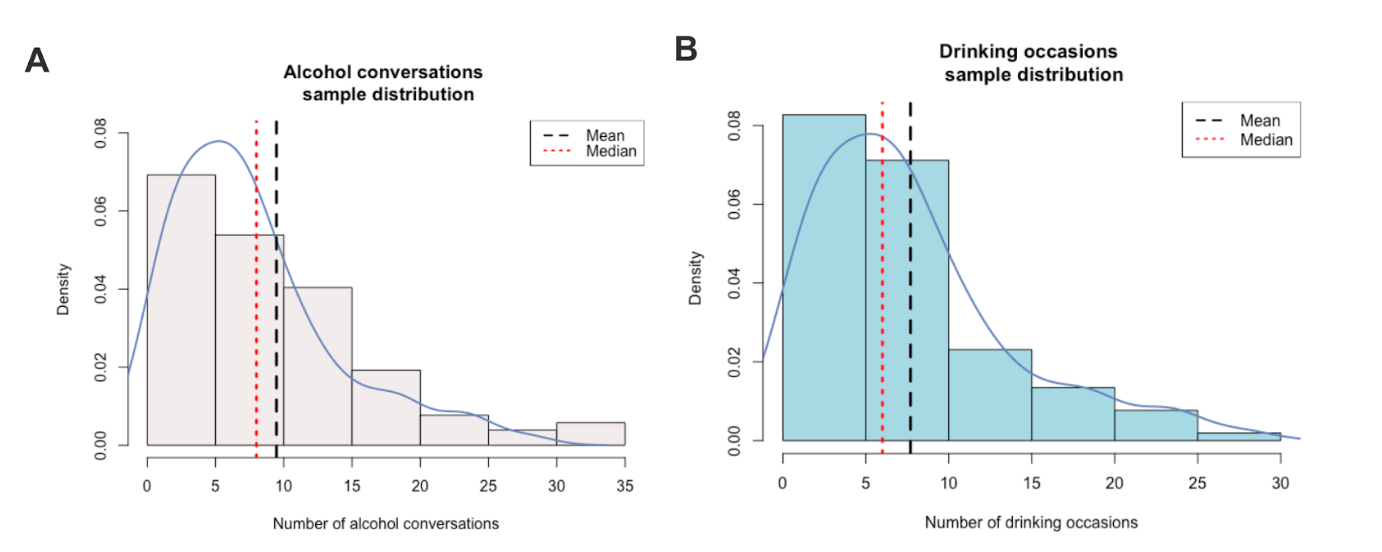
**

**Table S8. Reward responses to drinking (vs. non-drinking) peer faces moderate the association between alcohol conversation and next-day drinking using zero-inflated negative binomial multilevel hurdle models.**

|  | | **Reward ROI*alcohol conversation on next-day drinking** | | | | | | | | |
| --- | --- | --- | --- | --- | --- | --- | --- | --- | --- | --- |
|  | | | **Zero-inflated sub model** | | | | **Conditional sub model** | | | |
| Fixed Effects | | | OR | 95%CI | | *p* | OR | 95%CI | | *p* |
|  | Intercept  Alcohol conversation  Reward activity  Baseline drinking frequency  Baseline drinking amount  Gender  Age  Race  Social Group  Social Weekend  Proportion of alcohol conversations  Alcohol responses  Time in study  Condition mindful (vs. control)  Condition perspective (vs. control)  Active week (vs. control week)  Reward activity*alcohol conversation | | 12.63  0.69  1.16  0.77  0.91  0.88  0.84  0.86  0.98  0.84  0.09  1.02  1.01  1.00  1.00  0.84  0.34 | 5.90,26.97  0.56, 0.86  0.60, 2.21  0.69, 0.86  0.85, 0.97  0.66, 1.18  0.78, 0.92  0.77, 0.97  0.93,1.04  0.70,0.99  0.04,0.23  1.00,1.04  1.01,1.02  0.71-1.42  0.70,1.45  0.67,1.05  0.13,0.86 | | <.001***  .001**  .661  <.001***  .004*  .397  <.001***  .014*  .531  .042*  <.001***  .120  <.001***  .999  .979  .129  .022* | 2.60  1.25  0.99  1.08  1.13  0.80  1.03  1.03  1.05  1.21  1.21  1.00  0.99  0.81  0.88  0.98  1.90 | 1.42, 4.75  1.03, 1.52  0.57, 1.71  1.00, 1.17  1.08, 1.18  0.64, 0.99  0.97,1.09  0.94, 1.12  1.00, 1.09  1.03,1.43  0.65,2.26  0.98,1.02  0.99,1.00  0.63-1.04  0.67-1.15  0.79-1.22  0.80-4.54 | | .002*  .023*  .968  .062  <.001***  .037*  .352  .515  .044*  .021*  .552  .986  .007**  .099  .352  .867  .147 |
| Random effects | | | Variance | | SD | | Variance | | SD | |
| Intercept  Participant ID | | | .39 | | .20 | | .25 | | .50 | |
|  | | |  | |  | |  | |  | |

*Note*. 4747 observations. **p* ≤ .05, ***p* ≤ .01, ****p* ≤ .001. The zero inflated sub- model of the hurdle model estimates the probability of an extra zero (no alcohol use). As such, an odds ratio of less than 1 corresponds to a positive effect, i.e. more occasions of alcohol use.

**Table S9. Mentalizing responses to drinking (vs. non-drinking) peer faces moderate the association between alcohol conversation and next-day drinking using zero-inflated negative binomial multilevel hurdle models.**

|  | | **Mentalizing ROI*alcohol conversation on next-day drinking** | | | | | | | | |
| --- | --- | --- | --- | --- | --- | --- | --- | --- | --- | --- |
|  | | | **Zero-inflated sub model** | | | | **Conditional sub model** | | | |
| Fixed Effects | | | OR | 95%CI | | *p* | OR | 95%CI | | *p* |
|  | Intercept  Alcohol conversation  Mentalizing activity  Baseline drinking frequency  Baseline drinking amount  Gender  Age  Race  Social Group  Social Weekend  Proportion of alcohol conversations  Alcohol responses  Time in study  Condition mindful (vs. control)  Condition perspective (vs. control)  Active week (vs. control week)  Mentalizing activity*alcohol conversation | | 12.42  0.69  1.15  0.77  0.90  0.89  0.85  0.86  0.98  0.84  0.09  1.02  1.01  1.01  1.01  0.84  0.38 | 5.81, .26.55  0.56, 0.85  0.70, 1.89  0.69, 0.86  0.84, 0.97  0.66, 1.19  0.78, 0.92  0.77, 0.97  0.93,1.04  0.70,1.00  0.03,0.22  1.00,1.04  1.01,1.43  0.71,1.43  0.70,1.46  0.67,1.05  0.19, 0.78 | | <.001***  .001**  .568  <.001***  .004*  .419  <.001***  .014*  .555  .046*  <.001***  .121  <.001***  .963  .952  .120  .008* | 2.69  1.26  1.03  1.07  1.13  0.79  1.03  1.02  1.04  1.22  1.24  1.00  0.99  0.81  0.87  0.98  1.29 | 1.47, 4.89  1.04, 1.53  0.69, 1.53  0.99, 1.16  1.08, 1.18  0.64, 0.97  0.97, 1.09  0.94, 1.11  1.00,1.09  1.03,1.43  0.66,2.33  0.98,1.02  0.99,1.00  0.62,1.04  0.67-1.14  0.79,1.21  0.68,2.48 | | .001**  .017*  .899  .080  <.001***  .027*  .379  .579  .052  .020*  .509  .951  .009*  .095  .314  .861  .435 |
| Random effects | | | Variance | | SD | | Variance | | SD | |
| Intercept  Participant ID | | | .25 | | .50 | | .25 | | .50 | |
|  | | |  | |  | |  | |  | |

*Note*. 4747 observations. **p* ≤ .05, ***p* ≤ .01, ****p* ≤ .001. The zero inflated sub- model of the hurdle model estimates the probability of an extra zero (no alcohol use). As such, an odds ratio of less than 1 corresponds to a positive effect, i.e. more occasions of alcohol use.

**Table S10. Correlations and Descriptive Statistics of Key Variables.**

| **Variables** | **1** | **2** | **3** | **4** | **5** |
| --- | --- | --- | --- | --- | --- |
| **1. Activity in reward ROI** | - |  |  |  |  |
| **2. Activity in mentalizing ROI** | 0.79 | - |  |  |  |
| **3. Count of alcohol conversations**  **4. Count of drinking occasions** | -0.02  -0.05 | -0.05  -0.09 | -  0.53 | - |  |
| **5. Number of alcohol responses** | 0.004 | 0.001 | 0.14 | 0.07 | - |
| **Mean** | 0.07 | 0.12 | 9.46 | 7.69 | 51.03 |
| **Standard Deviation** | 0.24 | 0.31 | 7.59 | 6.07 | 7.2 |

*Note*: *N*=104

**Brain activity effects on alcohol conversations and next-day drinking are specific to alcohol conversations.** We conducted additional analyses to examine the specificity of alcohol-related conversations by replacing them with discussions about water and caffeine (e.g., “Since the previous survey (morning or evening), have you talked to someone about drinking water/drinking caffeine drinks?”). These analyses revealed no significant interaction effects between conversations about water or caffeine and brain activity on next-day drinking for either mentalizing or reward-related brain activity. Specifically, for water conversations, the interaction with mentalizing activity yielded an odds ratio (OR) of 1.25, 95% CI [0.29–5.48], *p* = 0.766, and the interaction with reward activity showed an OR of 1.53, 95% CI [0.21–11.29], *p* = 0.675. Similarly, caffeine conversations did not significantly interact with mentalizing (OR = 2.38, 95% CI [0.63–8.98], *p* = 0.200) or reward activity (OR = 3.22, 95% CI [0.49–21.30], *p* = 0.226) on next-day drinking. These findings strengthen our confidence that the main interactions reported in the manuscript are specific to alcohol-related conversations, as no comparable effects were observed for conversations on non-alcoholic drinks.

**Brain activity effects on alcohol conversations and next-day drinking are robust to concurrent drinking.** If the effects we focus on (conversations on day *t* related to drinking on day *t+1*), were primarily driven by people’s levels of drinking, we would expect that controlling for current day’s drinking (*t*) might wash away the main interaction effects reported in the manuscript. We conducted additional analyses to test whether the main interaction effects for both reward and mentalizing activity remained significant even after controlling for concurrent drinking. Individuals with stronger activity in these regions in response to drinking (vs. non-drinking) peers showed a stronger positive association between alcohol-related conversations and next-day drinking, even when controlling for concurrent drinking [mentalizing: OR=2.15, 95% CI [1.06–4.37], *p* = 0.034, reward regions: OR =2.72, 95% CI [1.08–6.88], *p* = 0.034. These results strengthen our confidence that even when accounting for drinking on the same day, the primary interaction between brain region activity, alcohol conversations, and next-day drinking remains robust.

**Brain activity effects and general peer closeness.** As a control test, we examined whether our findings are specific to brain responses to faces of peers with varying alcohol related peer interactions, versus brain responses tracking peer closeness more generally. Specifically, we created a neural index of activity to peer closeness during passive face viewing (i.e., viewing faces to peers who are perceived as more vs. less close, range 1-9). We extracted mean parameter estimates from the parametrically modulated viewing of peer faces within the reward and the mentalizing system ROIs. Greater values on these neural indices correspond to stronger activity to faces of peers who are perceived as close vs. distant.

We then repeated the same interaction analyses as those presented in the main manuscript, however replacing the peer drinking (vs. non-drinking) neural index with a peer close (vs. distant) neural index. We found no significant interaction between brain activity to peers who are more (vs. less) close and alcohol conversations on future alcohol use in the reward system (OR =1.19, 95% CI [0.98, 1.45], *p*=0.073) and in the mentalizing system (OR =1.11, 95% CI [0.89, 1.39], *p*=0.341). However, the reward interaction trended in the same direction as the main effects presented in the main manuscript. See Tables S11 and S12 for more details. These results suggest that the main findings presented in the manuscript are unlikely to be driven by individual differences in the brain that track peer closeness more generally. These results suggest that future research is needed to more precisely isolate the neural effects of peer closeness and drinking interactions and to better understand how each independently may interact with alcohol conversations on next-day drinking.

**Table S11. Reward ROI perceived peer closeness model**

| **Reward activity*alcohol conversation effects on next-day drinking** | | | | |
| --- | --- | --- | --- | --- |
|  | |  | | |
| Fixed Effects | | OR | 95%CI | *P* |
|  | Intercept  Alcohol conversation  Reward activity  Baseline drinking frequency  Baseline drinking amount  Proportion of alcohol conversations  Time in study  Alcohol conversation*reward activity | 0.18  1.39  0.98  1.35  1.07  11.69  0.99  1.19 | 0.14, 0.22  1.12, 1.73  0.84, 1.15  1.20, 1.52  1.00, 1.15  4.26, 32.09  0.98, 0.99  0.98, 1.45 | <.001***  .002**  .783  <.001***  .058  <.001***  <.001***  .073 |
|  | | ICC | SD | |
| Intercept  Participant ID | | .10 | .60 | |

*Note*. 4760 Observations. **p* ≤ .05, ***p* ≤ .01, ****p* ≤ .001. Reward activity is parametrically extracted to track perceived peer closeness (close vs. distant).

**Table S12. Mentalizing ROI perceived peer closeness model**

| **Mentalizing activity*alcohol conversation effects on next-day drinking** | | | | |
| --- | --- | --- | --- | --- |
|  | |  | | |
| Fixed Effects | | OR | 95%CI | *P* |
|  | Intercept  Alcohol conversation  Mentalizing activity  Baseline drinking frequency  Baseline drinking amount  Proportion of alcohol conversations  Time in study  Mentalizing activity*alcohol conversation | 0.18  1.45  0.95  1.34  1.07  11.80  0.99  1.11 | 0.14, 0.22  0.18, 1.78  0.78, 1.14  1.19, 1.51  1.00, 1.15  4.31, 32.31  0.98, 0.99  0.89, 1.39 | <.001***  <.001***  .566  <.001***  .058  <.001***  <.001***  .341 |
|  | | ICC | SD | |
| Intercept  Participant ID | | .10 | .60 | |

*Note.* 4760 Observations. **p* ≤ .05, ***p* ≤ .01, ****p* ≤ .001. Mentalizing activity is parametrically extracted to track perceived peer closeness (close vs. distant).

**Brain activity effects on alcohol conversations and next-day drinking are not driven by conversation valence.** We conducted additional analysis to explore if the main results presented in the main manuscript may be driven by how positively (vs. negatively) individuals talked about alcohol (a) at each time point, and (b) overall, across the 28 days.

Overall, conversations about alcohol were positive throughout the 28-day study protocol (*M* = 62.61, *SD*= 11.67, median = 61.6; range=32.35-91.33 from 0-100 scale). We found no significant interactions between brain activity and conversation valence on next-day drinking. Individual differences in brain responses to drinking (vs. non-drinking) peers did not significantly interact with conversation valence in the reward system, both within-person (OR = 0.99, 95% CI [0.94-1.04], *p* =0.562), and between-person (OR =1.03, 95% CI [0.97-1.109], *p* =0.352). We found similar non-significant effects for the mentalizing system both within-person (OR =0.99, 95% CI [0.95-1.03], *p*=0.723) and between-person (OR =1.05, 95% CI [1.00-1.09], *p*=0.051). Only the between-person interactions effects in the mentalizing system were marginal. Together, these results suggest the main results reported in the manuscript are less likely to be driven by the degree to which individuals perceived their alcohol-related conversations to be positive or negative.

**Supplementary References**

1. [Jovanova, M. *et al.* Psychological distance intervention reminders reduce alcohol consumption frequency in daily life. *Sci. Rep.* **13**, 12045 (2023).](http://paperpile.com/b/pU2wHm/KCbb)

2. [Cosme, D. *et al.* Study protocol: Social health impact of Network Effects (SHINE) study. *PsyArXiv* (2022) doi:](http://paperpile.com/b/pU2wHm/nAIG)[10.31234/osf.io/cj2nx](http://dx.doi.org/10.31234/osf.io/cj2nx)[.](http://paperpile.com/b/pU2wHm/nAIG)

3. [Esteban, O. *et al.* fMRIPrep: a robust preprocessing pipeline for functional MRI. *Nat. Methods* **16**, 111–116 (2019).](http://paperpile.com/b/pU2wHm/Wx4n7)

4. [Gorgolewski, K. *et al.* Nipype: a flexible, lightweight and extensible neuroimaging data processing framework in python. *Front. Neuroinform.* **5**, 13 (2011).](http://paperpile.com/b/pU2wHm/e87i)

5. [Tustison, N. J. *et al.* N4ITK: improved N3 bias correction. *IEEE Trans. Med. Imaging* **29**, 1310–1320 (2010).](http://paperpile.com/b/pU2wHm/IriCq)

6. [Avants, B. B., Epstein, C. L., Grossman, M. & Gee, J. C. Symmetric diffeomorphic image registration with cross-correlation: evaluating automated labeling of elderly and neurodegenerative brain. *Med. Image Anal.* **12**, 26–41 (2008).](http://paperpile.com/b/pU2wHm/twcZB)

7. [Zhang, Y., Brady, M. & Smith, S. Segmentation of brain MR images through a hidden Markov random field model and the expectation-maximization algorithm. *IEEE Trans. Med. Imaging* **20**, 45–57 (2001).](http://paperpile.com/b/pU2wHm/KVBtp)

8. [Dale, A. M., Fischl, B. & Sereno, M. I. Cortical surface-based analysis. I. Segmentation and surface reconstruction. *Neuroimage* **9**, 179–194 (1999).](http://paperpile.com/b/pU2wHm/k4CRP)

9. [Klein, A. *et al.* Mindboggling morphometry of human brains. *PLoS Comput. Biol.* **13**, e1005350 (2017).](http://paperpile.com/b/pU2wHm/hQim3)

10. [Fonov, V. S., Evans, A. C., McKinstry, R. C., Almli, C. R. & Collins, D. L. Unbiased nonlinear average age-appropriate brain templates from birth to adulthood. *Neuroimage* **Supplement 1**, S102 (2009).](http://paperpile.com/b/pU2wHm/VIdPn)

11. [Cox, R. W. & Hyde, J. S. Software tools for analysis and visualization of fMRI data. *NMR in Biomedicine* vol. 10 171–178 Preprint at https://doi.org/](http://paperpile.com/b/pU2wHm/Xy7zQ)[10.1002/(sici)1099-1492(199706/08)10:4/5<171::aid-nbm453>3.0.co;2-l](http://dx.doi.org/10.1002/(sici)1099-1492(199706/08)10:4/5%3C171::aid-nbm453%3E3.0.co;2-l) [(1997).](http://paperpile.com/b/pU2wHm/Xy7zQ)

12. [Greve, D. N. & Fischl, B. Accurate and robust brain image alignment using boundary-based registration. *Neuroimage* **48**, 63–72 (2009).](http://paperpile.com/b/pU2wHm/fTzUT)

13. [Jenkinson, M., Bannister, P., Brady, M. & Smith, S. Improved optimization for the robust and accurate linear registration and motion correction of brain images. *Neuroimage* **17**, 825–841 (2002).](http://paperpile.com/b/pU2wHm/L65zV)

14. [Lanczos, C. Evaluation of Noisy Data. *Journal of the Society for Industrial and Applied Mathematics Series B Numerical Analysis* **1**, 76–85 (1964).](http://paperpile.com/b/pU2wHm/sgcWm)

15. [Creators Cosme, Danielle1 Flournoy, John C. 2 Vijayakumar, Nandita3 Show affiliations 1. University of Oregon 2. Harvard University 3. Deakin University. *Auto-Motion-Fmriprep: A Tool for Automated Assessment of Motion Artifacts*. doi:](http://paperpile.com/b/pU2wHm/tgh9)[10.5281/zenodo.1412131](http://dx.doi.org/10.5281/zenodo.1412131)[.](http://paperpile.com/b/pU2wHm/tgh9)

16. [Corbin, N., Todd, N., Friston, K. J. & Callaghan, M. F. Accurate modeling of temporal correlations in rapidly sampled fMRI time series. *Hum. Brain Mapp.* **39**, 3884–3897 (2018).](http://paperpile.com/b/pU2wHm/HWnM)

17. [Penny, W. D., Friston, K. J., Ashburner, J. T., Kiebel, S. J. & Nichols, T. E. *Statistical Parametric Mapping: The Analysis of Functional Brain Images*. (Elsevier, 2011).](http://paperpile.com/b/pU2wHm/VbvyP)

18. [Yarkoni, T., Poldrack, R. A., Nichols, T. E., Van Essen, D. C. & Wager, T. D. Large-scale automated synthesis of human functional neuroimaging data. *Nat. Methods* **8**, 665–670 (2011).](http://paperpile.com/b/pU2wHm/aS0W)

19. [Nilearn. *Nilearn*](http://paperpile.com/b/pU2wHm/uLGJ8) <https://nilearn.github.io/stable/modules/generated/nilearn.plotting.plot_roi.html> [(2022).](http://paperpile.com/b/pU2wHm/uLGJ8)
